# Supplementary material for: Aerodigestive sampling reveals altered microbial exchange between lung, oropharyngeal, and gastric microbiomes in children with impaired swallow function
Source: PLoS One. 2019 May 20;14(5):e0216453. doi: 10.1371/journal.pone.0216453 (PMC6527209; doi:10.1371/journal.pone.0216453)
Supplement: S1 Table — (PDF) [file pone.0216453.s001.pdf]

	Aspiration/Penetration	Normal	Fisher's p
**Demographics**			
Gender	30 M, 17 F	31 M, 26 F	
Age	3.9 ± 4.1 years	6.7 ± 4.7 years	
**Symptom and quality of life scores**			
PGSQ symptom score	0.9 ± 0.75	0.9 ± 0.71	
PGSQ total score	0.7 ± 0.61	0.9 ± 0.70	
**Medications**			
Currently taking PPIs	55% (26/47)	58% (33/57)	0.84
Currently taking H2 blockers	15% (7/47)	21% (12/57)	0.46
Current use of inhaled steroids	72% (34/47)	75% (43/57)	0.82
**Symptoms within last 6 months**			
Problem swallowing	7% (3/41)	22% (12/54)	0.09
Food stuck	29% (12/41)	26% (14/54)	0.82
Difficulty swallowing	34% (14/41)	28% (15/54)	0.51
Abdominal pain	37% (16/43)	32% (18/56)	0.67
Constipation	35% (14/40)	37% (19/52)	1.00
Weight loss	22% (9/40)	30% (16/54)	0.49
Food coming up	30% (13/44)	39% (22/56)	0.40
Chest pain	10% (4/40)	24% (13/54)	0.11
Chronic cough	65% (30/46)	72% (38/53)	0.52
**Infection history within 6 months**			
History of pneumonia	33% (15/45)	33% (18/54)	1.00
Recent history of ear infection	42% (18/43)	19% (10/54)	0.01
Recent history of sinus infection	25% (11/44)	26% (14/53)	1.00
History of any recent antibiotics	28% (13/47)	39% (22/57)	0.30

Supplementary Table 1: Patient demographics, separated by aspiration status. While all patients were given questionnaires, not all patients completed the answers to all questions. Fisher's exact p-values were calculated on the contingency table of aspiration status and the metadata indicated in each row, and tests whether the distribution of symptoms, medications, or infection history is similarly distributed between aspirators and non-aspirators. For example, the Fisher's exact test for the "Currently on PPIs" column was calculated on the following contingency table: [[(47 - 26), 26], [(57 - 33), 33]].
