# Supplementary material for: Aerodigestive sampling reveals altered microbial exchange between lung, oropharyngeal, and gastric microbiomes in children with impaired swallow function
Source: PLoS One. 2019 May 20;14(5):e0216453. doi: 10.1371/journal.pone.0216453 (PMC6527209; doi:10.1371/journal.pone.0216453)
Supplement: S2 Table — (PDF) [file pone.0216453.s002.pdf]

	**Aspiration/penetration**	**Normal**	**Not tested**
BAL, gastric fluid, and oropharyngeal swab	23	19	24
BAL and gastric fluid	6	9	7
BAL and oropharyngeal swab	2	4	1
Gastric fluid and oropharyngeal swab	9	16	20
Oropharyngeal swab and stool			20
BAL only	2	1	3
Gastric fluid only	3	4	5
Oropharyngeal swab only	2	4	31
Stool only			5
Total patients	47	57	118

Supplementary Table 2: Number of patients with each combination of body sites sequenced, separated by aspiration status.
