# Supplementary material for: Aerodigestive sampling reveals altered microbial exchange between lung, oropharyngeal, and gastric microbiomes in children with impaired swallow function
Source: PLoS One. 2019 May 20;14(5):e0216453. doi: 10.1371/journal.pone.0216453 (PMC6527209; doi:10.1371/journal.pone.0216453)
Supplement: S3 Table — P-values associated with analysis presented in Fig 4. (PDF) [file pone.0216453.s003.pdf]

Within-patient sites	Between-patient site	p	Direction
Lung and oropharynx	lung	0.71	within $<$ between
Lung and oropharynx	oropharynx	2×10^{-8}	within $>$ between
Lung and gastric fluid	lung	9×10^{-12}	within $<$ between
Lung and gastric fluid	gastric fluid	7×10^{-8}	within $<$ between
Gastric fluid and oropharynx	gastric fluid	5×10^{-15}	within $<$ between
Gastric fluid and oropharynx	oropharynx	0.18	within $<$ between

Supplementary Table 3: **Lung and gastric microbial communities are driven primarily by person rather than body site.** We compared the within-patient JSD for all pairs of aerodigestive sites with the average across-patient JSD between each of the sites in the within-patient comparison. For each pair of aerodigestive sites, we compared each patient's within-patient JSD with the average across-patient JSD for each site in the pair. For example, the top row shows the comparisons between (1) the JSD between each patient's own oropharyngeal and lung communities ("Within-patient sites") with (2) the average JSD between that patient's lung community and all other lung communities ("Between-patient site"). We calculated Wilcoxon signed-rank p-values using Python's `scipy.stats.wilcoxon` function ("p"). The "Direction" column indicates whether the within-patient JSD was larger (i.e. more different) than the between-patient JSD (`within > between`), or vice-versa (`within < between`).
