# Supplementary material for: Aerodigestive sampling reveals altered microbial exchange between lung, oropharyngeal, and gastric microbiomes in children with impaired swallow function
Source: PLoS One. 2019 May 20;14(5):e0216453. doi: 10.1371/journal.pone.0216453 (PMC6527209; doi:10.1371/journal.pone.0216453)
Supplement: S4 Table — (PDF) [file pone.0216453.s004.pdf]

	Site	Minimum q-value
**Genus-level**	Lung	0.23
	Gastric fluid	0.35
	Oropharyngeal swab	0.27
**OTU-level**	Lung	0.60
	Gastric fluid	0.11
	Oropharyngeal swab	0.18

Supplementary Table 4: Differential abundance analysis comparing aspirators vs. non-aspirators yields no significant results. P-values were calculated on the relative abundances of genera and OTUs with the Kruskal-Wallis test implemented in Python's `scipy.stats.mstats` module (function `kruskalwallis`, a non-parametric test and an implementation which accounts for ties). P-values were corrected for multiple hypothesis testing with the `multipletests` function from `statsmodels.sandbox.stats.multicomp`, with the Benjamini/Hochberg correction (`method = 'fdr_bh'`). P-values were corrected separately for each site and each level of analysis (e.g. OTU or genus-level).
