# Supplementary material for: Aerodigestive sampling reveals altered microbial exchange between lung, oropharyngeal, and gastric microbiomes in children with impaired swallow function
Source: PLoS One. 2019 May 20;14(5):e0216453. doi: 10.1371/journal.pone.0216453 (PMC6527209; doi:10.1371/journal.pone.0216453)
Supplement: S5 Table — (PDF) [file pone.0216453.s005.pdf]

Family	Genus	Non-aspirator	Aspirator	Difference
Neisseriaceae	Neisseria	7.1	41.4	34.2
Porphyromonadaceae	Porphyromonas	28.6	62.1	33.5
Pasteurellaceae	Haemophilus	50.0	82.8	32.8
Lachnospiraceae	Coprococcus	10.7	37.9	27.2
Micrococcaceae	Rothia	14.3	41.4	27.1
Prevotellaceae	Prevotella	25.0	51.7	26.7
Carnobacteriaceae	Granulicatella	32.1	58.6	26.5
Bacillales_Incertae_Sedis_XI	Gemella	42.9	69.0	26.1
Pasteurellaceae	Haemophilus	57.1	82.8	25.6
Actinomycetaceae	Actinomyces	17.9	41.4	23.5
Streptococcaceae	Streptococcus	39.3	62.1	22.8
Lachnospiraceae	Oribacterium	14.3	34.5	20.2
Leptotrichiaceae	Streptobacillus	17.9	37.9	20.1
Lachnospiraceae	Lachnoanaerobaculum	17.9	37.9	20.1
Fusobacteriaceae	Fusobacterium	42.9	62.1	19.2
Prevotellaceae		50.0	69.0	19.0
Flavobacteriaceae	Planobacterium	14.3	31.0	16.7
Leptotrichiaceae	Leptotrichia	14.3	31.0	16.7
Erysipelotrichaceae	Solobacterium	17.9	34.5	16.6
Prevotellaceae	Prevotella	21.4	37.9	16.5
Pasteurellaceae	Haemophilus	28.6	44.8	16.3
Veillonellaceae	Veillonella	35.7	51.7	16.0
Enterobacteriaceae	Escherichia/Shigella	46.4	62.1	15.6
Prevotellaceae		46.4	62.1	15.6
Neisseriaceae	Neisseria	60.7	75.9	15.1
Streptococcaceae	Streptococcus	75.0	89.7	14.7
Veillonellaceae	Veillonella	35.7	48.3	12.6
Prevotellaceae	Prevotella	42.9	55.2	12.3
Micrococcaceae	Rothia	42.9	55.2	12.3
Streptococcaceae	Streptococcus	42.9	55.2	12.3
Prevotellaceae	Prevotella	64.3	75.9	11.6
Unknown Burkholderiales		10.7	20.7	10.0
Bacteroidaceae	Bacteroides	14.3	24.1	9.9
Porphyromonadaceae	Porphyromonas	21.4	31.0	9.6
Moraxellaceae	Moraxella	39.3	48.3	9.0
Prevotellaceae	Prevotella	57.1	65.5	8.4
Leptotrichiaceae	Leptotrichia	21.4	27.6	6.2
Fusobacteriaceae	Fusobacterium	25.0	31.0	6.0
Porphyromonadaceae	Porphyromonas	50.0	55.2	5.2
Neisseriaceae	Neisseria	17.9	20.7	2.8
Veillonellaceae	Veillonella	89.3	89.7	0.4
Coriobacteriaceae	Atopobium	21.4	20.7	-0.7
Unknown Bacteria		21.4	20.7	-0.7
Enterococcaceae		85.7	82.8	-3.0
Chloroplast	Streptophyta	10.7	6.9	-3.8
Pasteurellaceae	Haemophilus	17.9	13.8	-4.1
Unknown Bacillales		17.9	13.8	-4.1
Unknown Bacillales		17.9	13.8	-4.1
Lactobacillaceae	Lactobacillus	28.6	17.2	-11.3
Pasteurellaceae	Haemophilus	32.1	20.7	-11.5
Staphylococcaceae	Staphylococcus	60.7	48.3	-12.4
Bacteroidaceae	Bacteroides	17.9	3.4	-14.4
Comamonadaceae	Acidovorax	17.9	3.4	-14.4
Porphyromonadaceae	Parabacteroides	21.4	6.9	-14.5
Comamonadaceae	Pelomonas	21.4	6.9	-14.5
Flavobacteriaceae	Chryseobacterium	28.6	13.8	-14.8
Erysipelotrichaceae	Clostridium_XVIII	21.4	3.4	-18.0
Lachnospiraceae	Ruminococcus2	25.0	6.9	-18.1
Flavobacteriaceae	Chryseobacterium	50.0	31.0	-19.0
Neisseriaceae	Microvirgula	57.1	37.9	-19.2
Enterobacteriaceae	Enterobacter	82.1	62.1	-20.1
Mycobacteriaceae	Mycobacterium	28.6	6.9	-21.7
Moraxellaceae	Acinetobacter	60.7	37.9	-22.8
Streptococcaceae	Streptococcus	60.7	37.9	-22.8
Bacteroidaceae	Bacteroides	53.6	27.6	-26.0
Unknown Bacillales		57.1	31.0	-26.1
Moraxellaceae	Acinetobacter	60.7	34.5	-26.2
Moraxellaceae	Enhydrobacter	60.7	34.5	-26.2
Lactobacillaceae	Lactobacillus	50.0	20.7	-29.3
Aeromonadaceae	Aeromonas	57.1	27.6	-29.6
Moraxellaceae	Acinetobacter	78.6	41.4	-37.2
Leuconostocaceae	Weissella	78.6	37.9	-40.6
Moraxellaceae	Acinetobacter	78.6	37.9	-40.6
Leuconostocaceae	Leuconostoc	78.6	37.9	-40.6
Streptococcaceae	Lactococcus	78.6	37.9	-40.6
Streptococcaceae	Lactococcus	78.6	37.9	-40.6

Supplementary Table 5: Prevalence of lung-gastric fluid exchanged OTUs. Prevalence is calculated as the percentage of patients who have the OTU present in both their lungs and oropharynx, calculated separately among aspirators (N = 29) and non-aspirators (N = 28). OTUs are ordered by their differential prevalence in aspirators relative to non-aspirators, and are labeled with their family- and genus-level taxonomies. Blank genus names indicate OTUs which were not annotated at the genus level.
