# Supplementary material for: Aerodigestive sampling reveals altered microbial exchange between lung, oropharyngeal, and gastric microbiomes in children with impaired swallow function
Source: PLoS One. 2019 May 20;14(5):e0216453. doi: 10.1371/journal.pone.0216453 (PMC6527209; doi:10.1371/journal.pone.0216453)
Supplement: S6 Table — (PDF) [file pone.0216453.s006.pdf]

**Lung-oropharynx OTUs (13)**	AUC	p	N (non-asp/asp)
Lung	0.59	0.21	33/33
Oropharyngeal	0.65	0.11	43/36
Both	0.70	0.08	23/25
**Lung-gastric OTUs (76)**			
Lung	0.56	0.45	33/33
Gastric fluid	0.66	0.03	48/41
Both	0.69	0.008	28/29

Supplementary Table 6: **Classifiers based on the abundance of exchanged OTUs.** (Top) Classifiers built from the abundance of lung-oropharynx exchanged OTUs. (Bottom) Classifiers built from the abundance of lung-gastric exchanged OTUs. Rows indicate which microbial community was used to train each classifier. In classifiers using two sites ("Both"), abundances of each exchanged OTU in each site were considered as separate features. AUCs are calculated as the area under the average ROC curve from leave-one-out predictions. Fisher's exact p values are calculated on the leave-one-out predictions using Python's `scipy.stats.fisher_exact` function.
