# Supplementary material for: Aerodigestive sampling reveals altered microbial exchange between lung, oropharyngeal, and gastric microbiomes in children with impaired swallow function
Source: PLoS One. 2019 May 20;14(5):e0216453. doi: 10.1371/journal.pone.0216453 (PMC6527209; doi:10.1371/journal.pone.0216453)
Supplement: S1 Fig — (PDF) [file pone.0216453.s007.pdf]

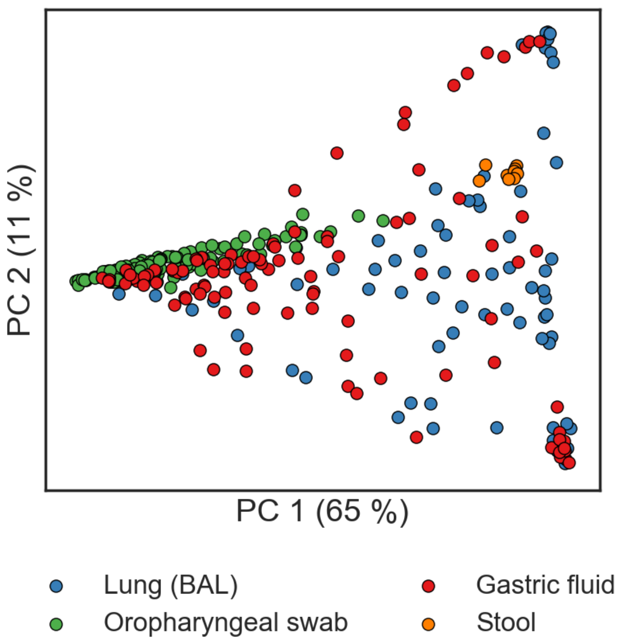


Supplementary Figure 1: PCoA plot of aerodigestive and stool microbial communities for patients in the sequencing batch not shown in the main text (N = 81 BAL, 124 oropharyngeal swab, 104 gastric fluid, and 11 stool samples). PERMANOVA on the BAL, gastric fluid, and oropharyngeal swab samples, $p = 0.001$.
