# Supplementary material for: Aerodigestive sampling reveals altered microbial exchange between lung, oropharyngeal, and gastric microbiomes in children with impaired swallow function
Source: PLoS One. 2019 May 20;14(5):e0216453. doi: 10.1371/journal.pone.0216453 (PMC6527209; doi:10.1371/journal.pone.0216453)
Supplement: S2 Fig — (PDF) [file pone.0216453.s008.pdf]

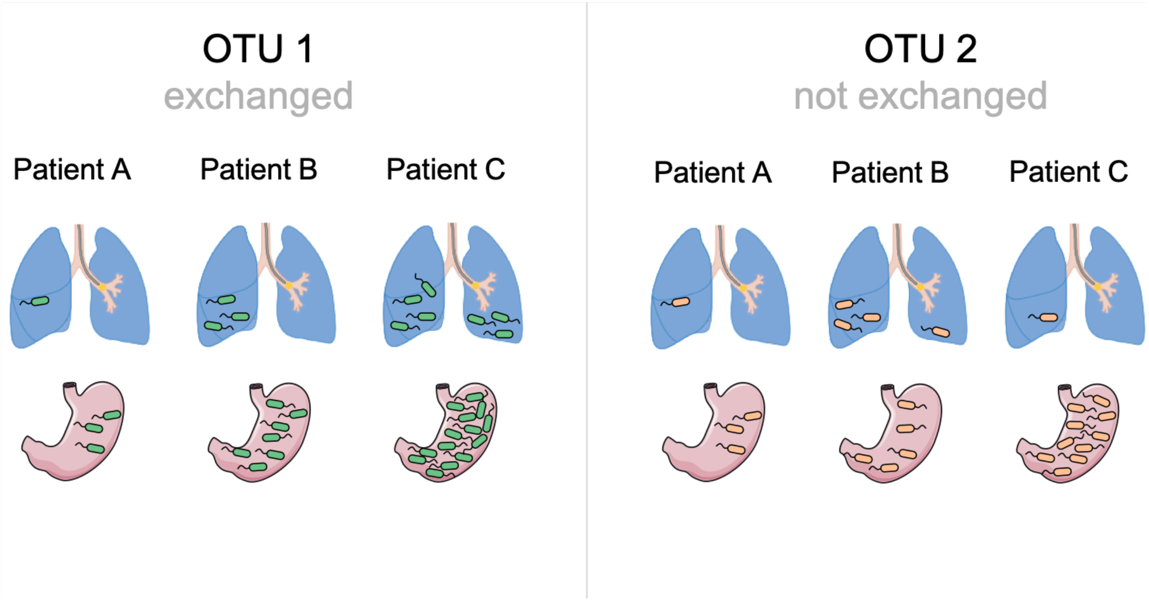

Supplementary Figure 2: Schematic illustrating an OTU which is considered exchanged between the lung and stomach (left) and one which is not (right). If an OTU is exchanged in two sites, its abundance in the two sites should be correlated across patients. For example, OTU 1 is exchanged across the lung and stomach. If its abundance in the stomach of Patient C is higher than in the stomach of Patient B, we expect that its abundance in the lungs of Patient C will be higher than in the lungs of Patient B. In contrast, OTU 2 is not exchanged, so knowing its abundance in Patient C's stomach relative to Patient B does not provide information about OTU 2's expected abundance in the lungs of Patient C. Lung image was adapted from Cancer Research UK / Wikimedia Commons and the stomach image is from Servier Medical Art.
