# Supplementary material for: Aerodigestive sampling reveals altered microbial exchange between lung, oropharyngeal, and gastric microbiomes in children with impaired swallow function
Source: PLoS One. 2019 May 20;14(5):e0216453. doi: 10.1371/journal.pone.0216453 (PMC6527209; doi:10.1371/journal.pone.0216453)
Supplement: S3 Fig — (PDF) [file pone.0216453.s009.pdf]

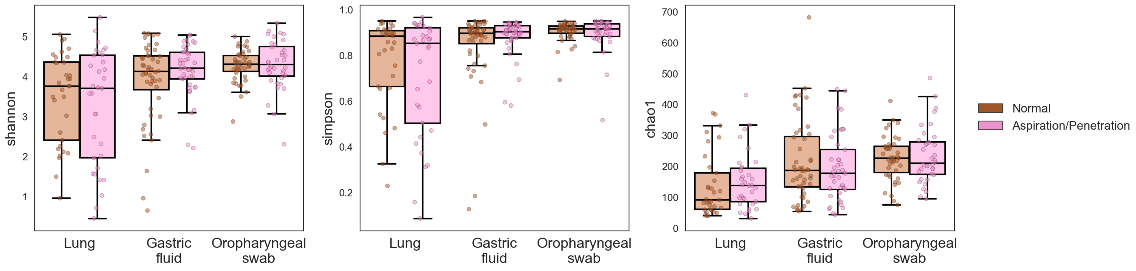

Supplementary Figure 3: Alpha diversity of each aerodigestive site compared between aspirators and non-aspirators. Each panel is a different metric, calculated with the respective metric (labeled on the y-axis) in `skbio.diversity.alpha`. All p-values for aspirator vs. non-aspirator comparisons are greater than 0.1 (Wilcoxon rank sums test calculated with Python's `scipy.stats.ranksums` function).
