# Supplementary material for: Aerodigestive sampling reveals altered microbial exchange between lung, oropharyngeal, and gastric microbiomes in children with impaired swallow function
Source: PLoS One. 2019 May 20;14(5):e0216453. doi: 10.1371/journal.pone.0216453 (PMC6527209; doi:10.1371/journal.pone.0216453)
Supplement: S4 Fig — (PDF) [file pone.0216453.s010.pdf]

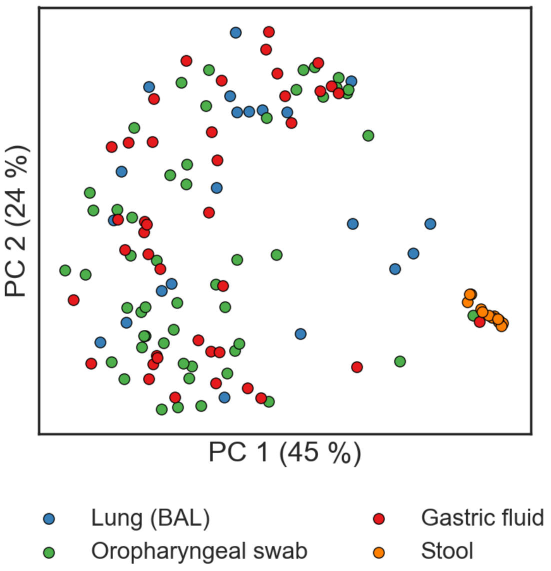

Supplementary Figure 4: PCoA plots of aerodigestive and stool microbial communities for all patients in the sequencing batch shown in Figure 2A, based on the Bray-Curtis distance.
