# Supplementary material for: Aerodigestive sampling reveals altered microbial exchange between lung, oropharyngeal, and gastric microbiomes in children with impaired swallow function
Source: PLoS One. 2019 May 20;14(5):e0216453. doi: 10.1371/journal.pone.0216453 (PMC6527209; doi:10.1371/journal.pone.0216453)
Supplement: S6 Fig — (PDF) [file pone.0216453.s012.pdf]

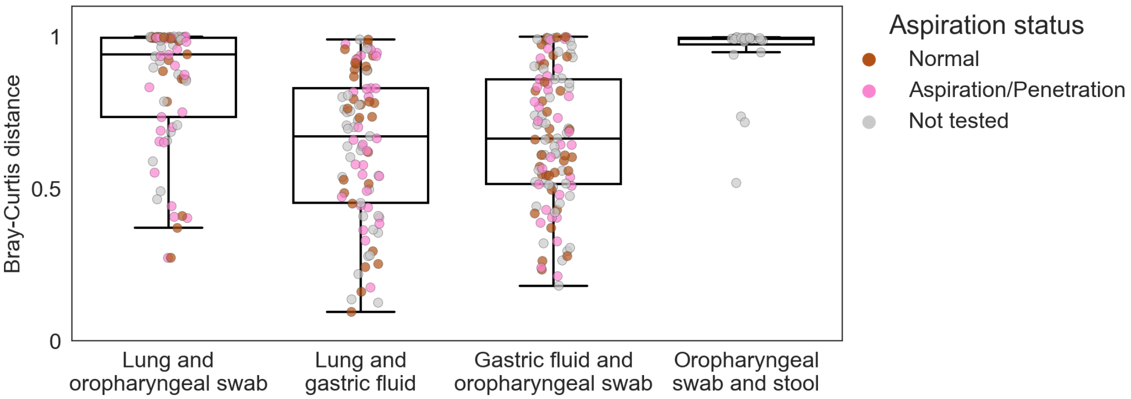

Supplementary Figure 6: Bray-Curtis distances between samples from different sites from the same patient. Comparisons between stool and oropharynx are included to contextualize these results, as these are expected to be very different. All comparisons are significant (Wilcoxon rank sums test calculated with Python's `scipy.stats.ranksums` function) except the lung and gastric fluid vs. gastric fluid and oropharyngeal swab beta diversities (p = 0.5) and the lung and oropharyngeal vs. oropharyngeal and stool (p = 0.2). All other comparisons: $p < 1 \times 10^{-6}$.
