# Supplementary material for: Aerodigestive sampling reveals altered microbial exchange between lung, oropharyngeal, and gastric microbiomes in children with impaired swallow function
Source: PLoS One. 2019 May 20;14(5):e0216453. doi: 10.1371/journal.pone.0216453 (PMC6527209; doi:10.1371/journal.pone.0216453)
Supplement: S7 Fig — (PDF) [file pone.0216453.s013.pdf]

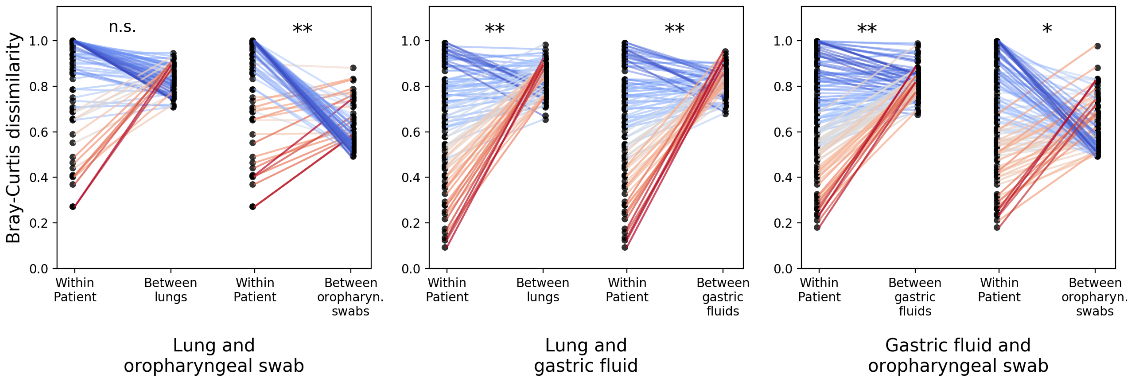

Supplementary Figure 7: Comparison between within-patient and between-patient beta diversities, as in Figure 4), calculated with the Bray-Curtis dissimilarity. $** : 10^{-10} < p < 10^{-6}$; $* : p = 0.04$.
