# Supplementary material for: Aerodigestive sampling reveals altered microbial exchange between lung, oropharyngeal, and gastric microbiomes in children with impaired swallow function
Source: PLoS One. 2019 May 20;14(5):e0216453. doi: 10.1371/journal.pone.0216453 (PMC6527209; doi:10.1371/journal.pone.0216453)
Supplement: S8 Fig — (PDF) [file pone.0216453.s014.pdf]

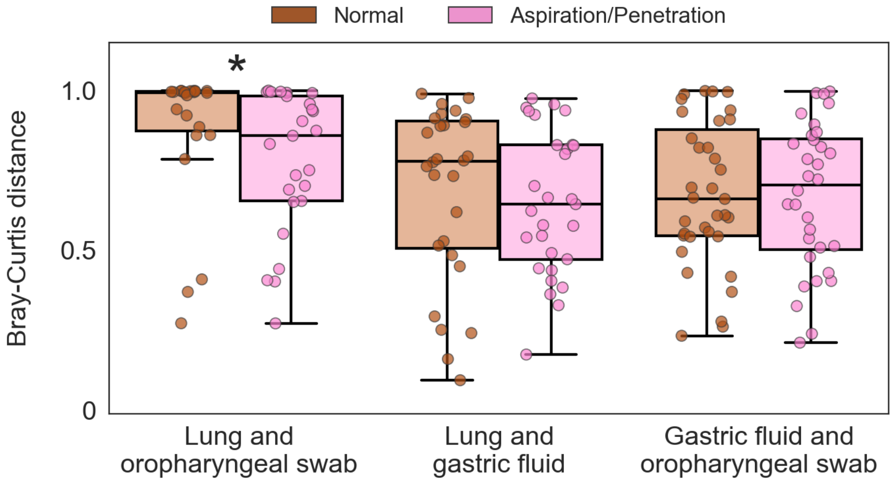

Supplementary Figure 8: Intra-patient Bray Curtis distance for different aerodigestive site comparisons in non-aspirators (brown) and aspirators (pink). Each point represents one patient. P-values (Wilcoxon rank sums test, calculated with Python's `scipy.stats.ranksums` function): lung and oropharyngeal swab $p = 0.02$, lung and gastric fluid $p = 0.5$, gastric fluid and oropharyngeal swab $p = 0.9$.
