# Supplementary material for: Aerodigestive sampling reveals altered microbial exchange between lung, oropharyngeal, and gastric microbiomes in children with impaired swallow function
Source: PLoS One. 2019 May 20;14(5):e0216453. doi: 10.1371/journal.pone.0216453 (PMC6527209; doi:10.1371/journal.pone.0216453)
Supplement: S9 Fig — (PDF) [file pone.0216453.s015.pdf]

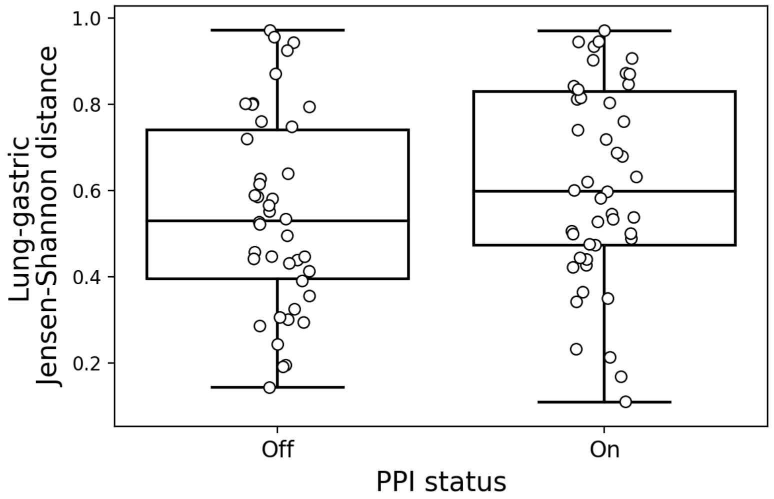

Supplementary Figure 9: Lung-gastric JSD vs. PPI status. Off PPI, N = 42; On PPI, N = 41. Wilcoxon rank sums test, calculated with Python's `scipy.stats.ranksums` function, p = 0.14.
