# Supplementary figures and images for: Aerodigestive sampling reveals altered microbial exchange between lung, oropharyngeal, and gastric microbiomes in children with impaired swallow function
Source: PLoS One. 2019 May 20;14(5):e0216453. doi: 10.1371/journal.pone.0216453 (PMC6527209; doi:10.1371/journal.pone.0216453)

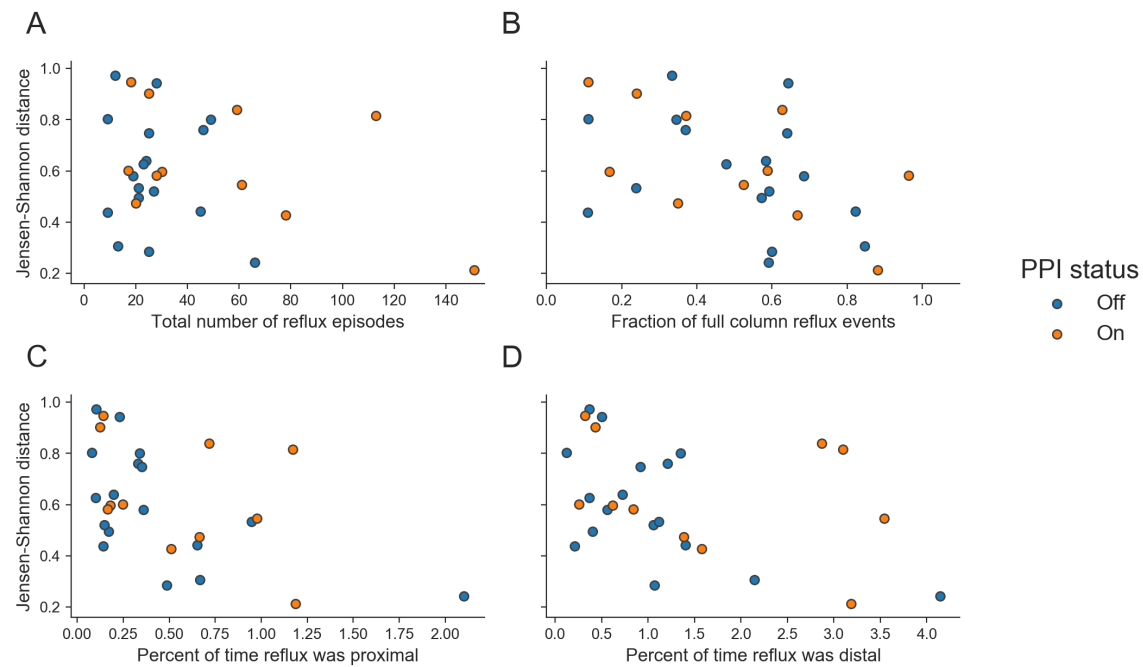

Supplementary Figure 10: Same data as in Figure 6, but points are colored by PPI status.

Supplement: S10 Fig — (PDF) [file pone.0216453.s016.pdf]
