# Supplementary material for: Aerodigestive sampling reveals altered microbial exchange between lung, oropharyngeal, and gastric microbiomes in children with impaired swallow function
Source: PLoS One. 2019 May 20;14(5):e0216453. doi: 10.1371/journal.pone.0216453 (PMC6527209; doi:10.1371/journal.pone.0216453)
Supplement: S11 Fig — (PDF) [file pone.0216453.s017.pdf]

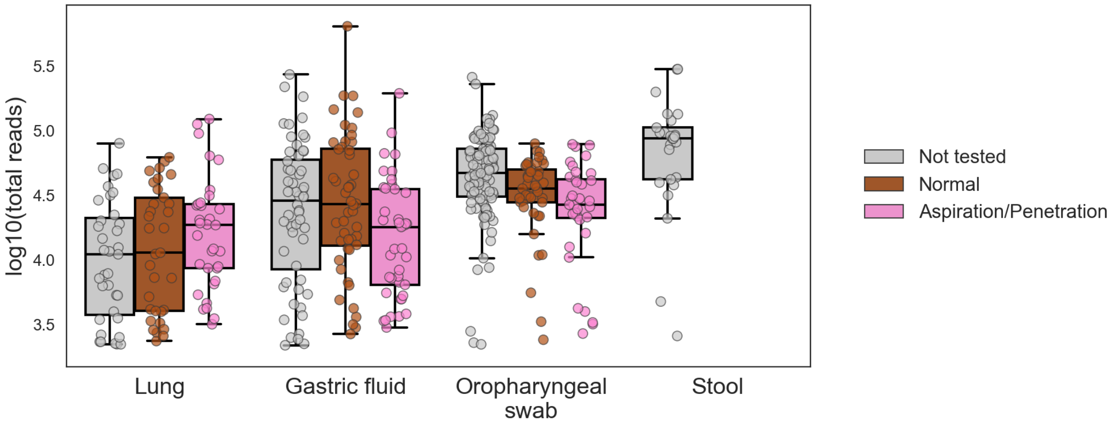

Supplementary Figure 11: Total reads per sample. P-values (Wilcoxon rank sums test, calculated with Python's `scipy.stats.ranksums` function) for aspiraton vs. non-aspirator comparison: lung $p = 0.3$, gastric fluid $p = 0.02$, oropharyngeal swab $p = 0.08$.
